# Employing optical lightning data to identify lightning flashes associated to terrestrial gamma-ray flashes

**DOI:** 10.1007/s42865-024-00065-y

**Published:** 2024-04-01

**Authors:** Christoph Köhn, Matthias Heumesser, Olivier Chanrion, Victor Reglero, Nikolai Østgaard, H. J. Christian, T. J. Lang, R. J. Blakeslee, Torsten Neubert

**Affiliations:** 1 National Space Institute (DTU Space), Technical University of Denmark, Kgs. Lyngby, Denmark; 2 Processing Laboratory, University of Valencia, Valencia, Spain; 3 Birkeland Centre for Space Science, University of Bergen, Bergen, Norway; 4 Department of Atmospheric Science, Earth System Science Center, University of Alabama in Huntsville, Huntsville, USA; 5 NASA Marshall Space Flight Center, Huntsville, USA

**Keywords:** ISS LIS, ASIM, TGFs

## Abstract

Terrestrial gamma-ray flashes (TGFs) are bursts of energetic X- and gamma-rays emitted from thunderstorms. The Atmosphere-Space Interactions Monitor (ASIM) mounted onto the International Space Station (ISS) is dedicated to measure TGFs and optical signatures of lightning; ISS LIS (Lightning Imaging Sensor) detects lightning flashes allowing for simultaneous measurements with ASIM. Whilst ASIM measures $\sim$300-400 TGFs per year, ISS LIS detects $\sim 10^6$ annual lightning flashes giving a disproportion of four orders of magnitude. Based on the temporal evolution of lightning flashes and the spatial pattern of the constituing events, we present an algorithm to reduce the number of space-detected flashes potentially associated with TGFs by $\sim$ 60% and of associated LIS groups by $\sim$ 95%. We use ASIM measurements to confirm that the resulting flashes are indeed those associated with TGFs detected at approx. 400 km altitude and thus benchmark our algorithm preserving 70–80% of TGFs from concurrent ASIM-LIS measurements. We compare how the radiance, footprint size and the global distribution of lightning flashes of the reduced set relates to the average of all measured lightning flashes and do not observe any significant difference. Finally, we present a parameter study of our algorithm and discuss which parameters can be tweaked to maximize the reduction efficiency whilst keeping flashes associated to TGFs. In the future, this algorithm will hence be capable of facilitating the search for TGFs in a reduced set of lightning flashes measured from space.

## Introduction

Terrestrial gamma-ray flashes (TGFs) are bursts of energetic X- and gamma-rays with photon energies of up to 40 MeV, which are emitted from thunderstorms and typically last for tens to hundreds of microseconds (Fishman 1994). They were first observed by the Burst and Transient Source Experiment (BATSE) (Fishman 1994), and subsequently reported by various other missions such as AGILE (Astro-Rivelatore Gamma a Immagini Leggero) (Marisaldi et al. 2010; Tavani et al. 2011), RHESSI (Reuven Ramaty High Energy Solar Spectroscopic Imager) (Cummer et al. 2005; Smith et al. 2010)and Fermi (Briggs et al. 2010) estimating that there are up to $\sim$ 400000 TGFs per year, or approx. 1100 per day (Briggs et al. 2013). TGFs are subject to the current ASIM (Atmosphere-Space Interactions Monitor) mission (Neubert et al. 2019) launched to the International Space Station (ISS) in April 2018 which provided several measurements of terrestrial gamma-ray flashes and concurrent lightning observations (Neubert et al. 2019; Østgaard et al. 2019a, b; Neubert et al. 2020; Heumesser et al. 2021; Lindanger et al. 2021) suggesting that at least some TGFs are emitted by propagating lightning leaders and the associated streamer-leader system (Köhn et al. 2020; Heumesser et al. 2021).

Lightning detection from space started in the mid 1990s, briefly after the first detection of TGFs, with the Optical Transient Detector (OTD) (Christian et al. 1996; Boccippio et al. 2000) and continued with the Lightning Imaging Sensor (LIS) on the Tropical Rainfall Measuring Mission (TRMM) satellite (Christian et al. 1999; Ushio et al. 2001). The groundbreaking results from these early missions led to the Geostationary Lightning Mapper (GLM), a commercial instrument aboard the GOES satellites, and the design of new lightning imagers (LI) for the third generation of meteorological (MTG) satellites in Europe (Stuhlmann et al. 2005).

A flight spare of the LIS instrument flown on TRMM was adapted to be flown again on the ISS. The ASIM mission started in early 2018 and since June 2018 LIS and ASIM make complementary measurements. Their simultaneous detection of optical emissions from lightning is a great synergy and allowed various insights (Blakeslee et al. 2020). However, ISS LIS measures approximately $10^6$ lightning flashes per year which can be downloaded through https://ghrc.nsstc.nasa.gov/pub/lis/iss/data/science/ (Lang 2022), whilst ASIM detects approximately 300-400 TGFs per year (Østgaard et al. 2019b), hence approx. four orders of magnitude less than the number of observed lightning flashes; when assuming 400000 TGFs per year (Briggs et al. 2013), only every second LIS flash should be associated with a TGF. Subsequently, when searching for TGFs in available lightning data, it is desirable to significantly decrease the number of lightning flashes to reduce the processing time. Additionally, commercial lightning imagers currently operate only in the 777 nm wavelength band, such as LIS, whereas ASIM records in multiple wavelength bands and has high-energy detectors. Hence, based on LIS data, we have developed an algorithm to identify space-detected lightning flashes, potentially associated with TGFs and to reveal more insights into their global occurrence rates as well as serving as a blueprint for the data product development of meteorological service providers such as EUMETSAT. The presented algorithm could potentially be used as a cross trigger for other detectors on the same satellite or detectors on ground. A possible second application for the algorithm is to generate probability distributions of TGFs based on the lightning flashes measured from satellites. In addition to space-based measurements of lightning and TGFs, there is a variety of past and present aircraft missions and measurements (Smith et al. 2011; Kochkin et al. 2015, 2018) such as the “Airborne Lightning Observatory for FEGS and TGFs” (ALOFT, FEGS stands for “Fly’s Eye Geostationary Lightning Mapper (GLM) Simulator”) (Østgaard et al. 2023a); although the presented algorithm is tailored to observations from space, it might serve as a prototype for an algorithm at thundercloud altitudes.

We briefly describe both instruments as well as the functionality of our algorithm in Section 2. In Section 3, we discuss the validity of the algorithm, present a parameter study for the designed algorithm as well as a statistical overview of the found TGFs and discuss how lightning flashes producing TGFs differ from those of average lightning flashes. We conclude in Section 4.

## Instrumentation and methodology

### Instrumentation

The development of the presented algorithm is based on space data, both for the lightning flashes and the analyzed TGFs. The lightning data used in this paper is provided by NASA’s ISS LIS detecting lightning at a wavelength of 777.4 nm, a spectral line of atomic oxygen, hence indicating hot lightning leaders. The optically sensitive CCD of LIS has $128\times 128$ pixels with 1 nm bandwidth, $\sim 2$ ms time resolution and a field of view of $80\times 80$ degrees^2^. LIS has a ground resolution of $4\times 4$ km^2^ at nadir and $6\times 6$ km^2^ at the edge of the field of view (FOV). Additionally, LIS detects lightning during the whole day with varying sensitivity due to the variation of cloud albedo during the day and night (Blakeslee et al. 2020). LIS data is divided into events, groups and flashes. Events occur when a single CCD pixel is triggered within a time window of $\sim 2$ ms integrating all the radiation reaching ISS LIS within this temporal cycle. Multiple events occurring in the same integration cycle are then collected into a group. The next clustering unit are flashes which is a collection of groups that lie within a spatial window of 5.5 km^2^ on the ground and within a time interval of 330 ms. Details of the LIS algorithm can be found in Mach et al. (2007).

TGFs are detected by the Modular X- and Gamma-ray Sensor (MXGS) of the Atmosphere-Space Interactions Monitor (ASIM), also mounted onto the ISS (Østgaard et al. 2019a; Neubert et al. 2019). MXGS consists of a low-energy (LED) cadmium zinc telluride (CZT) detector, measuring photons with energies between approx. 15 and 400 keV and a high-energy (HED) Bismuth Germanate (BGO) detector for single photon energies of $\sim 0.2-30$ MeV with a time resolution of $\lesssim 1\ \mu$s. The two detectors are linked to a data processing unit (DPU) monitoring the incidence of photon bursts which defines the trigger time. In case of an incident photon, a trigger signal will be generated and all events within a 2-second time interval around the trigger time will be stored (Østgaard et al. 2019a). MXGS has a cross-trigger mode with the Modular Multi-spectral Imaging Array (MMIA) with a time resolution of 1 $\mu$s (Østgaard et al. 2019a) detecting optical signatures of lightning in the wavelengths 180-230 nm, 337 nm and 777 nm (Chanrion et al. 2019). The relative timing accuracy between MMIA and MXGS amounts to approx. 10 $\mu$s.

**Table 1:** The temporal and spatial resolution as well as the field of view (FOV) and the number of pixels for the ISS LIS instrument as well as for the low-energy (LED) and high-energy (HED) detector of MXGS

Instrument	Temporal resolution	Spatial resolution (ISS LIS)/	FOV size	Pixel
		Location accuarcy (MXGS)		
ISS LIS	2 ms	4 km $\times$ 4 km (nadir)	$80^{\circ } \times 80^{\circ }$	$128\times 128$
		6 km $\times$ 6 km (edge of FOV)		
MXGS LED	1 $\mu$s	$$\left. \begin{array}{l} \text {Point source:}<0.7^{\circ } \\ 3^{\circ }\ \text {diffuse source:} <2.0^{\circ } \end{array} \right\} $$	$80^{\circ } \times 80^{\circ }$	$128\times 128$
MXGS HED	28.7 ns		–	–

Note that HED works with non-pixelated BGO crystals. The location accuracy is valid for the whole MXGS instrument

Table 1 summarizes and compares the detector characteristics of ISS LIS and MXGS onboard ASIM. The number of pixels and the FOV size is the same for ISS LIS and for the LED; the HED, consisting of BGO bars, is not pixelated, simply measuring the quantum energy of photons. The temporal resolution of ISS LIS is larger than of MXGS, however sufficient to individually detect lighting events, groups and flashes. The low time resolution for MXGS is certainly needed to measure the fast bursts of energetic TGF photons, but not for measuring the lightning flashes; hence a direct comparison of these two temporal resolutions would be misleading. Note that the MMIA cameras onboard ASIM (detecting optical signals of streamers and leaders) have temporal resolutions in the order of ms, thus comparable to ISS LIS.

### Algorithm design

In this section, we discuss and present the algoritm to reduce the number of LIS flashes potentially associated with TGFs. Within one year LIS detects approx. $10^6$ flashes, corresponding to $\approx 10^7$ groups. The presented algorithm is decreasing the number of flashes and groups by approximately 60% and 95%, respectively, see Section 3 for dicussion. It has two consecutive steps: In the first step (see Section 2.2.1), potential groups of those LIS flashes associated with TGFs are selected based on their temporal evolution and the ratio between the main activity and the pre-activity. This is based on the fact that TGFs predominantly occur at the onset of streamer and leader activity (Heumesser et al. 2021; Köhn et al. 2020). Secondly, the events of such a “candidate group” are used to restore their spatial pattern (see Section 2.2.2) which is then used to refine the number of candidate groups and thus of flashes potentially associated with TGFs. Flash, group and event characteristics might depend on cloud characteristics, the altitude of a lightning flash or the position of the lightning flash in the field of view of LIS; however, these properties do not change the relative timing of groups within a flash or the relative ratio between the pre-activity and the main peak of these groups as the detection geometry will stay the same since TGFs do not occur at cloud tops as previous studies showed (Heumesser et al. 2021; Tiberia et al. 2021). There might be a slight influence of cloud or lightning characteristics on the spatial pattern of the LIS events (step 2); nonetheless, as we show in Sections 3.1 and 3.2, this does not make the algorithm mal-functioned, but still allows to reduce the number of LIS flashes and groups while keeping the majority of flashes potentially associated with TGFs.

The parameter set used in the next two subsections is rather found empirically, based on previous work (Gjesteland et al. 2017; Larkey et al. 2019; Alnussirat et al. 2019; Heumesser et al. 2021; Alnussirat et al. 2023). Additionally, we perform a parameter study in Section 3.1 showing that the presented algorithm is robust and working.

**Fig. 1:**
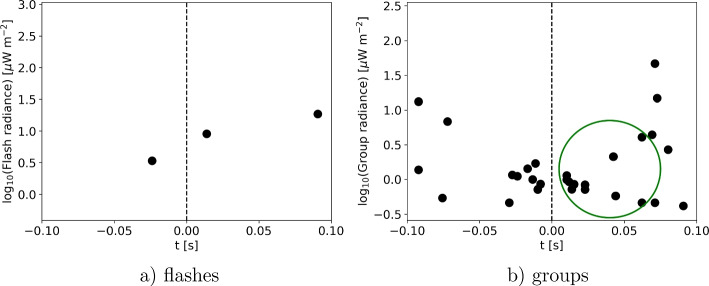
a) The radiance of LIS flashes as a function of time on August, 14th, 2019 . b) The radiance of LIS groups constituing these LIS flashes. The green circle depicts the first 11 groups of the first LIS flash after $t=0$ used for the algorithm marking the ASIM trigger time of the associated TGF at 13:03:21.024409 UTC (dashed line)

#### Select a candidate group

Since TGFs are reported to occur at the beginning of lightning flashes, i.e. at the onset activity of streamers and leaders (Heumesser et al. 2021), it is sufficient to limit the search for potential TGFs to the start of every LIS flash. We here use a time interval of $t_{f,0}=16.2$ ms in the beginning of each flash corresponding to a number of 9-11 groups, see Fig. 1; however, only a maximum of $max_g=9$ groups is used. This value for $t_{f,0}$ originates from a timing analysis from TRMM LIS, also valid for ISS LIS (Bitzer and Christian 2015). The minimum group integration times are 1.495 ms and 1.511 ms whilst the maximum integration times are 1.999 or 2.014 ms. Subsequently, the maximum time interval for 9 groups amounts to (1.511 ms $\cdot 4$ + 2.014 ms $\cdot 5$) $\approx$ 16.2 ms where we consider that approx. one half of the groups has a timing of 1.511 ms and the other one 2.014 ms. This time also corresponds to the earliest observed pre-activity for TGFs in the 777 nm band (Heumesser et al. 2021; Heumesser 2021). This is in alignment with work presented by Alnussirat et al. (2023) who found that TGFs occur at the onset of the optical activity. Additionally, their analysis has revealed (see their Fig. 4) that the majority of flashes associated with TGFs has $\lesssim$ 10 groups (“strokes” in their terminology) which validates our choice of $max_g=9$. Note that this (and the following) parameter(s) are to some extent empirical parameters; we will present a parameter study in Section 3.1 showing that these values are suitable for the presented algorithm and that the algorithm efficiency does not depend significantly on the parameters described here.

The selected 9 groups (as indicated by the green circle in Fig. 1b) are then tested for their consecutive occurrence. This is important since the detected emissions of longer pulses can be split into one or more groups and the possible pre-activity has to be distinguished from the main pulse. TGFs usually occur together with the main peak (Heumesser et al. 2021), hence we only want to pass the group of the main peak to the next algorithm step (concerning the spatial distribution of LIS events) and thus we need to discard pre-activity. To do so, the selection of groups is split into blocks of directly consecutive times. This means that all groups which are directly adjacent in time are considered as one block. If only one such block exists, it is taken further. If more such blocks occur, the groups in the first block are counted. Pre-activity has distinct pulse durations that are not longer than 2 LIS groups; hence blocks with more groups are not considered pre-activity. The maximum temporal separation associated with these 2 LIS groups, i.e. between the end of pre-activity and the beginning of the main pulse, amounts to approximately $\Delta t_{pre-main}=5.6$ ms (Heumesser et al. 2021). Further, this pre-activity can maximally occur up to 6.042 ms (3 integration cycles of 2.014 ms duration) before the next block of consecutive groups, which would represent the main pulse in this scenario. Last, (Heumesser et al. 2021) have shown that the 75^th^ percentile of the ratio between pre-activity and main pulse of 777 flashes (see their Fig. S2 and Table S3) amounts to approx. 22%; hence, for the algorithm we have chosen that the pre-activity intensity has to be below $r=22\%$ of the intensity of the next block. If all those checks are passed, the first block is considered pre-activity and consequently, the second block is selected. Otherwise, the first block is used to proceed.

The selected block must not consist of more than $max_b=4$ groups, since the observed optical pulses by ASIM in connection to TGFs were always within this time (Heumesser et al. 2021).

The current algorithm does not include any sort of threshold for the detected LIS radiance, so the $max_b=4$ groups account for the maximal spread of pulse durations over multiple LIS groups. A block of consecutive groups that passes all criteria above is considered to be the optical representation of a flash potentially associated with a TGF. The group with the highest radiance of this block is then selected for the second step of the algorithm because it most likely represents the whole or at least the maximum of the peak.

#### Determine spatial pattern of events

**Fig. 2:**
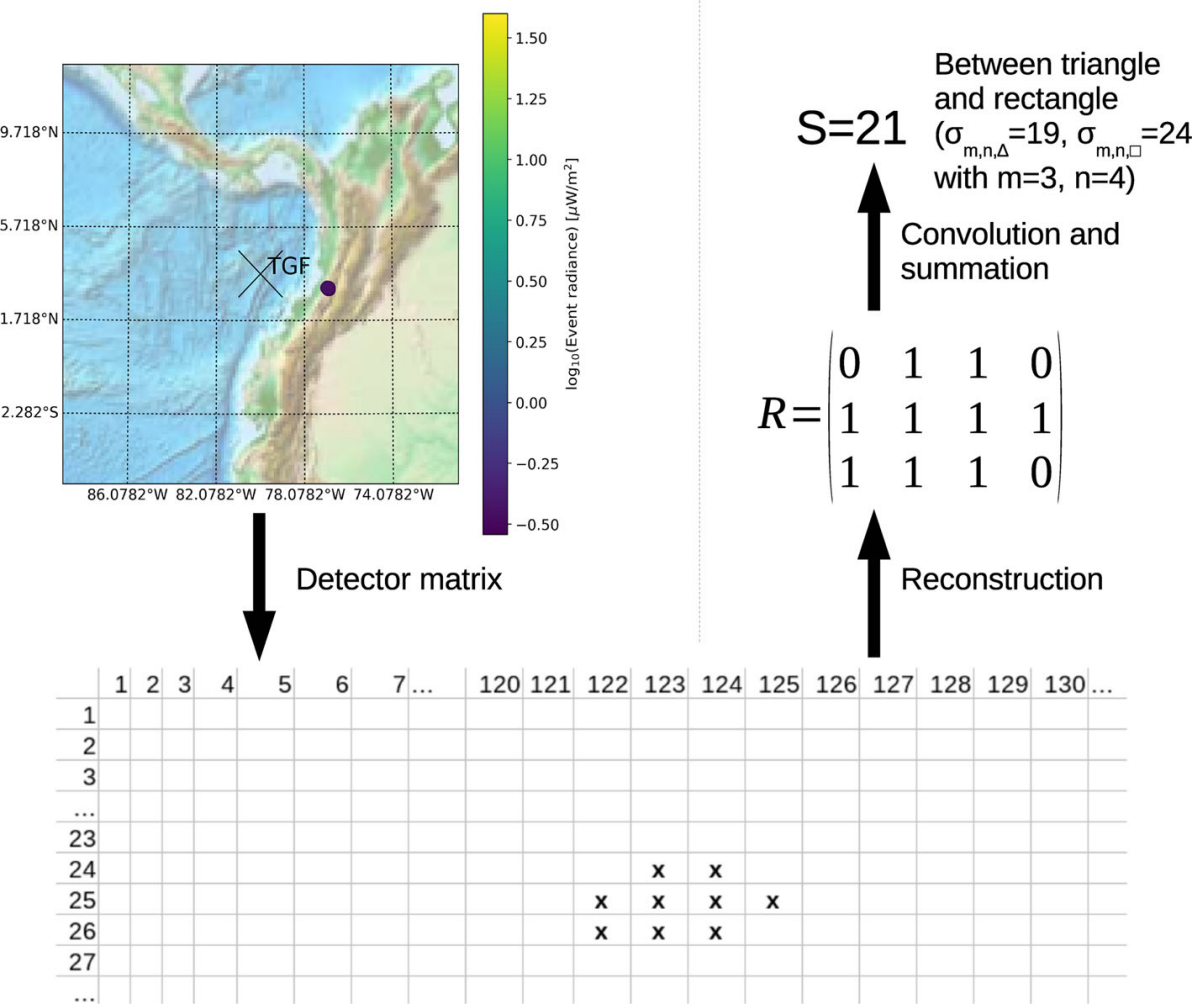
The algorithm to determine the spatial pattern of LIS events: The position of the LIS events of a certain group (top left) is mapped to the detector matrix with integer indices for columns and rows (bottom). This matrix pattern defines a new matrix *R* (1) consisting of as many rows and columns as the maximum extention in the detector matrix (mid right); a 1 corresponds to a “**x**” in the detector matrix, otherwise a 0 is set. Finally, the sum *S* of the matrix convolution (2) is calculated to determine the spatial pattern as summarized in Table 2. This example is for a TGF on April, 12th, 2019, at 01:48:04.361385 UTC

The events constituting the group selected in the first step are now taken and their original detection pattern is restored. This is necessary since the events are stored without any direct information about their neighbours. Note that one pixel detection events are discarded since they are either noise or, even if a real signal, do not give much information about the group constituing events. Since TGF producing flashes are mainly moving upwards (Stanley et al. 2006; Marshall et al. 2013; Lyu et al. 2015, 2016; Cummer et al. 2015; Stolzenburg et al. 2016; Heumesser et al. 2021), under optimal observation geometry and cloud conditions, TGF related emissions form a circular pattern. However, since the detector matrix itself has a rectangular geometry (see Fig. 2), we would expect squares as the main spatial pattern for flashes associated with TGFs. Finally, we take into account that signals might be at the edge of FOV of the detector or that the radiance at the edge might be weak such that we weaken the condition of quadratic spatial patterns to triangular ones. Additionally, we allow for the slanting of the signal modifying quadratic signals into rectangular ones and all signals in-between. Since this information is relevant to detect potential TGFs, we here discuss how to restore and discriminate the spatial pattern of the events. Coordinates of LIS events are given as two-dimensional indices which form the rows and columns of the detector matrix (Fig. 2). As a first step, the spatial distribution of all group events is reconstructed by defining a matrix of zeros and ones

1 $$\begin{aligned} R=\left. \underbrace{\left( \begin{array}{cccc} 1 &{} 0 &{} \ldots &{} 0 \\ 0 &{} 1 &{} \ldots &{} 0 \\ \vdots &{} \ddots &{} \ddots \vdots \\ 0 &{} 1 &{} \ldots &{} 0 \end{array} \right) }_{i\ \text {elements}} \right\} j\ \text {elements} \end{aligned}$$

where *i* and *j* index the different two-dimensional coordinates of all LIS events of the target group between the maximum and minimum row and column of the detector matrix. *R* is designed such that $(i,j)=(1,1)$ corresponds to the minimum row and column indices of the event entries in the detector matrix ((24,122) in the example demonstrated in Fig. 2). A matrix entry is set to 1 when there is any event for a given coordinate set (*i*, *j*) (which corresponds to a “**x**” in the detector matrix); otherwise it is set to 0. In order to identify a distinct spatial pattern, e.g. rectangular or triangular, the minimum size of *R* needs to be $2\times 2$, i.e. we need at least 2 events in different rows or column. Considering upwards moving lightning flashes (Stanley et al. 2006; Marshall et al. 2013; Lyu et al. 2015, 2016; Cummer et al. 2015; Stolzenburg et al. 2016; Heumesser et al. 2021), we do not except big extensions of the spatial pattern; hence we impose that the size of *R* should not exceed 6 rows or columns. Additionally, we require that $|j-i |\le 2$ to assure an approximate shape between triangular and rectangular. These three conditions are found empirically, but make sense in the context of circular detection patterns and slight distortions thereof. If a pattern meets these criteria, a convolution with a $2\times 2$ kernel of ones is performed. The latter is useful to find the shape of a pattern as a specific value of the sum *S* of all the entries of the convolved matrix sum, hence of

2 $$\begin{aligned} S=\sum \limits _{k,l} \left( R *\left( \begin{array}{cc} 1 &{} 1 \\ 1 &{} 1 \end{array} \right) \right) _{k,l} \end{aligned}$$

**Table 2:** Matrix sums $\sigma _{m,n}$ for different shapes depending on the number *m* of rows and the number *n* of columns of the reconstruction matrix *R* (1)

Shape	Matrix sums $\sigma _{m,n}$
Rectangle	$4\cdot (m-1)(n-1)$
Cornerless rectangles	$4\cdot (m-1)(n-1)-4$
Triangle	$3\left( \min (m,n)-1\right) +\min (m,n)-2 +4\cdot \sum \limits _{j=1}^{\min (m,n)-1} j$

An explanation of these sums is given in Appendix A

where $*$ denotes the convolution of matrices. The sum *S* is compared with particular matrix sums $\sigma _{m,n}$ of the matrix (*m*, *n*) matrix *R* (1) for rectangles, rectangles without the corner pixels ("cornerless rectangles") and triangles, summarized in Table 2 and outlined in Appendix A. A triangular shape has the least amount of events while square or rectangles have the largest, accordingly for their sums. As we have argued above, to pass the selection criterion for the LIS events, including slanting, weak signals or signals at the edge of the FOV, all shapes with values between triangles and rectangles/squares are allowed, i.e.

3 $$\begin{aligned} \sigma _{m,n,\triangle }\le S\le \sigma _{m,n,\Box }. \end{aligned}$$

If the sum does not match any of the pre-defined shapes exactly, but is within the allowed limits, it is classified as “other”. An example of how the spatial pattern is determined is shown in Fig. 2 for a TGF on April, 12th, 2019. If the spatial pattern of events passes this final selection, the considered group is a TGF producing candidate and the block of groups as well as the flash, to which these groups belong to, are considered to potentially occur in connection to a TGF.

**Fig. 3:**
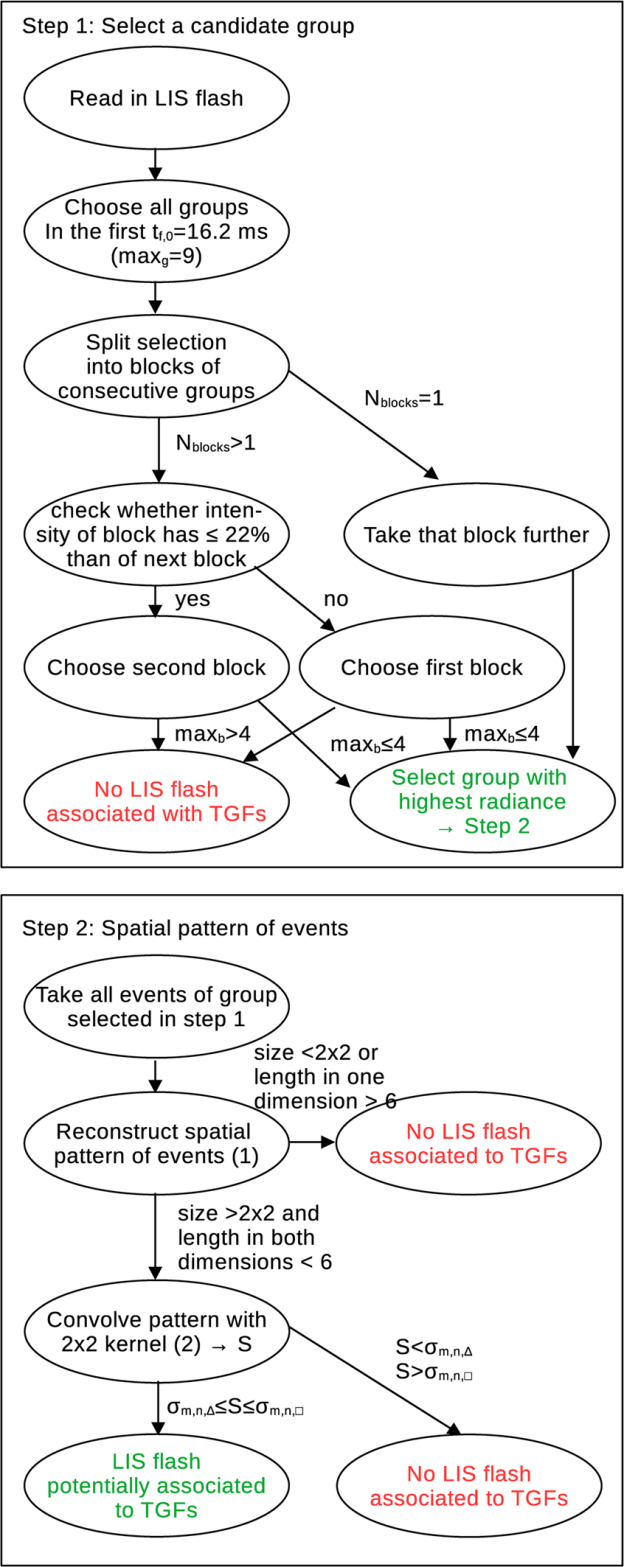
Schematic overview of the algorithm design finding LIS flashes potentially associated with TGFs. *m* and *n* are the number of rows and columns of the reconstruction matrix *R* (1); $\sigma _{m,n,\triangle /\Box }$ is the matrix sum for triangular and rectangular shape summarized in Table 2. The justification for the algorithm is described thoroughly in Sections 2.2.1 and 2.2.2

Figure 3 summarizes the algorithm to determine groups and flashes potentially associated with TGFs. We then define the reduction rate

4 $$\begin{aligned} SR_{fl,gr}=\frac{N_{total,fl/gr}-N_{cand,fl/gr}}{N_{total,fl/gr}}=1-\frac{N_{cand,fl/gr}}{N_{total,fl/gr}} \end{aligned}$$

where $N_{total,fl/gr}$ is the total number of LIS flash (fl) or group (gr) detections for a given year and $N_{cand,fl/gr}$ the number of LIS flashes or groups potentially associated with TGFs, calculated by the algorithm depicted in Fig. 3.

#### Number of TGFs in the reduced dataset

Whilst the algorithm described in Sections 2.2.1 and 2.2.2 reduces the number of LIS flashes and groups, it is vice-versa not self-evident that all the TGFs measured by ASIM in a given year coincide in space and time with the flashes of the reduced dataset. In order to determine coincident measurements of TGFs with LIS flashes of the reduced dataset, we calculate the time difference

5 $$\begin{aligned} \Delta t_{TGF-LIS}=|t_{TGF}-t_{flashes}| \end{aligned}$$

between the trigger time $t_{TGF}$ of each TGF per year and all the times $t_{flashes}$ of all flashes of the reduced dataset at the same day as a given TGF. Because of the absolute time uncertainty, ASIM detects events up to 34 ms earlier compared to other sources (Heumesser 2021). We thus consider a TGF concurrent with a LIS flash if $\Delta t_{TGF-LIS}\le 34$ ms. If 34 ms $<\Delta t_{TGF-LIS}\le 100$ ms, we say that this is a potential TGF-LIS coincidence. In Section 3.2, we will discuss how sensitively the number of coincident TGFs and LIS flashes depends on $\Delta _{TGF-LIS}$. Note that we check our algorithm against TGFs measured at ISS altitude which favours high-fluence events. The dataset measured by ASIM might only be a small subsample of all TGFs, part of them absorbed during their propagation up to $\sim 400$ km altitude (Smith et al. 2010; Nisi et al. 2014; Østgaard et al. 2023b). However, as the presented algorithm uses lightning flashes detected by ISS LIS, we believe that such a subsample might be appropriate for validating the algorithm.

#### Algorithm code

The code can be downloaded from

https://gitlab.gbar.dtu.dk/chrstk/lis-tgf-algorithm.git Provided LIS and ASIM data are available, it can subsequently be run locally on a laptop or desktop machine. Details of the code are summarized in Appendix B.

**Fig. 4:**
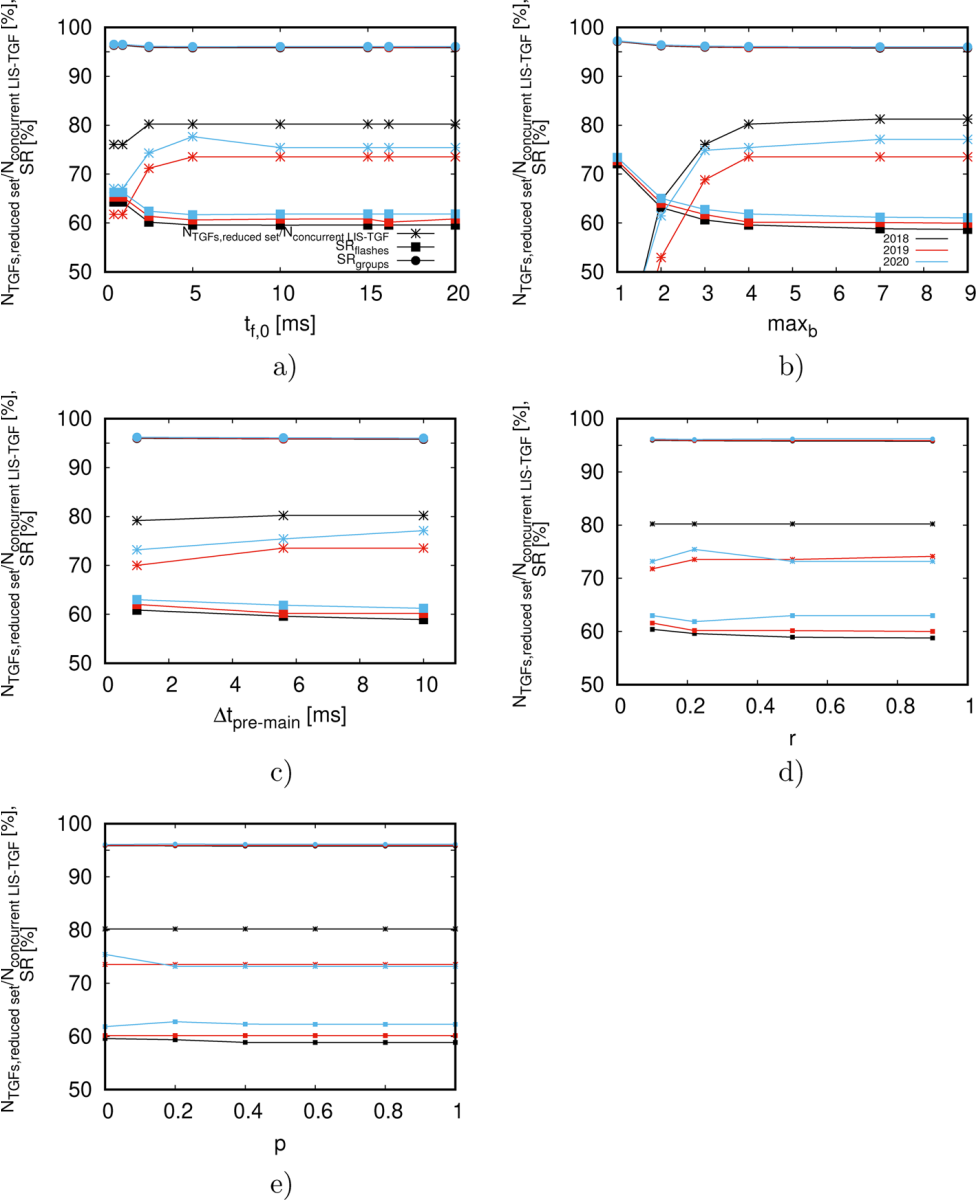
a)-d) The percentage $N_{\text {TGFs,reduced set}}/N_{\text { concurrent LIS-TGF}}$ of TGFs per year associated with the LIS flashes of the reduced dataset relative to the total number of concurrent LIS-TGF measurements (lines with stars) as well as the reduction efficiencies $SR_{flashes}$ (squares) and $SR_{groups}$ (circles) depending on the group parameters $t_{f,0}$, $max_b$, $\Delta t_{pre-main}$ and *r*. Black lines show data for 2018, red ones for 2019 and blue ones for 2020. e) $N_{\text {TGFs,reduced set}}/N_{\text { concurrent LIS-TGF}}$, $SR_{flashes}$ and $SR_{groups}$ as a function of *p* when adjusting condition Eq. 3 to $\sigma _{m,n,\triangle }(1-p)\le S\le \sigma _{m,n,\Box }(1+p)$

## Results

### Sensitivity of data reduction efficiency on group parameters and the spatial pattern of events

As mentioned in Section 2.2.1, the algorithm to select a potential candidate group depends on time interval $t_{f,0}$ in the beginning of each flash, on the number $max_b$ of groups per block, the maximum temporal separation $\Delta t_{pre-main}$ between the pre-activity and main peak as well as the maximum ratio *r* between the intensity of the pre-activity and of the main peak. As outlined in Section 2.2.1, the default set to run the algorithm is $t_{f,0}=16.2$ ms, $max_b=4$, $\Delta t_{pre-main}=5.6$ ms and $r=0.22$. For the years 2018–2020, Fig. 4 shows the reduction rate $SR_{fl,gr}$ Eq. 4 as a function of $t_{f,0}$ (a), $max_b$ (b), $\Delta t_{pre,main}$ (c) and *r* (d). For all of these panels, the non-varied parameters are set to the aforementioned default values. For all considered years, it shows that in most cases the reduction rate $SR_{fl}$ varies around 60% whilst $SR_{gr}$ is in the order of 95%. Panels a), c) and d) show that $SR_{fl,gr}$ is rather insensitive to $t_{f,0}$, $\Delta t_{pre-main}$ and *r*. There is only a slight increase up to $\approx 65\%$ when $t_{f,0}\rightarrow 0$. The most significant dependence is on $max_b$; decreasing $max_b$ will increase $SR_{fl}$ from approx. 60% to 72%. However, as we discuss in the next section, this implies also a lower number of coincident LIS-TGF events for the reduced dataset which is why we keep using $max_b=4$ for the algorithm.

Panel e) shows how $SR_{fl,gr}$ depends on the convolution sum condition Eq. 3. We found that $SR_{fl,gr}$ does not change significantly when weakening Eq. 3 to $\sigma _{m,n,\triangle }(1-p)\le S\le \sigma _{m,n,\Box }(1+p)$ for values of $p\in [0,1]$. For $p=0$, this equality reduces to Eq. 3 and we have chosen a maximum of $p=1$ such that the left hand side of the inequality does not become negative. Additionally, we investigated how $SR_{fl,gr}$ changes when completely turning off the dependence on the spatial pattern, i.e. not applying Eq. 3. Whilst $SR_{gr}$ reduces to approx. 90%, hence does not change significantly, $SR_{fl}$ decreases from $\sim 60\%$ to approx. $2-3\%$, thus worsening the algorithm considerably. Hence, it is not sufficient to only check the temporal evolution of the groups as described in Section 2.2.1, but the algorithm needs to use the spatial pattern as described in Section 2.2.2.

**Table 3:** The total number of TGFs per year detected by ASIM (first row), the number of concurrent LIS and TGF measurements (second row), the number of TGFs associated with flashes and groups of the reduced LIS datasets, i.e. $\Delta t_{TGF-LIS}\le 34$ ms (third row) as well as the number of potential TGFs associated with LIS flashes of the reduced dataset for 34 ms $<\Delta t_{TGF-LIS}\le 100$ ms (fourth row) and 34 ms $<\Delta t_{TGF-LIS}\le 200$ ms (fifth row)

	2018	2019	2020	2021
Total no. of TGFs	172	314	348	351
Concurrent LIS-TGF measurements	96	170	179	207
No. of TGFs associated	77 (80%)	125 (74%)	135 (75%)	146 (71%)
to LIS flashes in reduced datasets				
Potential TGF candidates	2 (2%)	14 (8%)	13 (7%)	16 (8%)
for 34 ms $<\Delta t_{TGF-LIS}\le 100$ ms				
Potential TGF candidates	7 (7%)	28 (16%)	27 (15%)	26 (13%)
for 34 ms $<\Delta t_{TGF-LIS}\le 200$ ms				

The number in brackets gives the percentage to the total number of concurrent LIS-TGF measurements (summarzied in the second row)

**Fig. 5:**
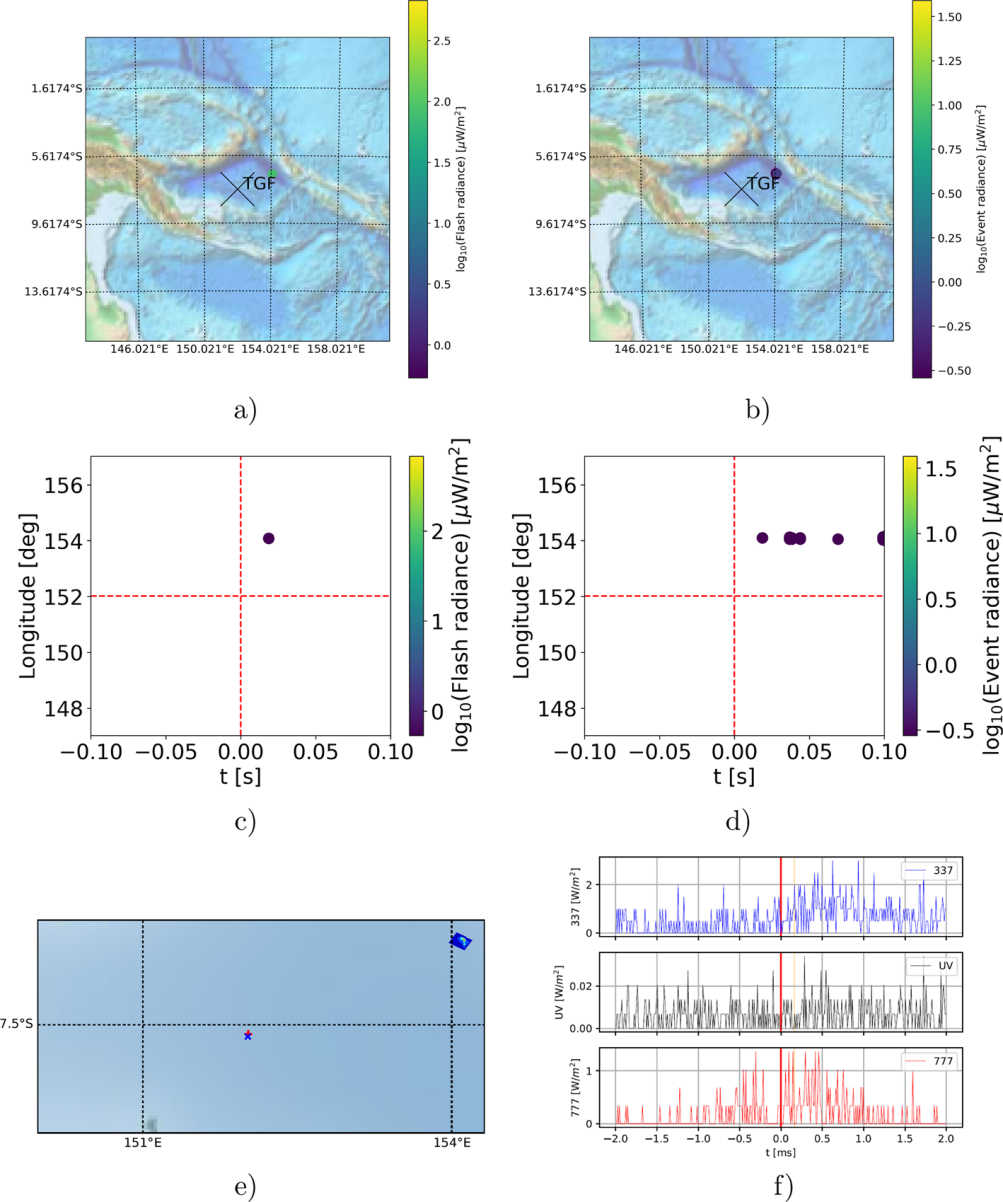
Example of a lightning event emitting a TGF, but not identified by the algorithm on Jan, 5th, 2019, 12:30:02.641575 UTC: a,b) Spatial distribution of the flashes (a) and events (b) detected by LIS, the cross markes the TGF position detected by ASIM; c,d) temporal evolution of LIS flashes (c) and events (d); e) optical data measured by ASIM (blue cross = footpoint of MXGS, red plus = footprint of MMIA); f) photometer signals measured by ASIM (t=0 is the TGF detection time) in the 337 nm (first row), 180-230 nm (second row) and 777 nm (third row) band

### Number of TGFs in the reduced dataset

The lines with stars in Fig. 4 display the ratio $N_{\text {TGF, reduced set}}/N_{\text {concurrent LIS-TGF}}$ of TGFs associated with the flashes of the reduced dataset and the total number of concurrent LIS and ASIM TGF measurements per year which are summarized in Table 3. Figure 4 shows that the algorithm keeps sufficiently many LIS flashes such that approx. 70-80% of the concurrent LIS and TGF measurements can be preserved. It also shows that - as for the reduction rate $SR_{fl,gr}$ - this ratio does not change significantly as a function of the algorithm parameters discussed in the previous section. The most important parameter is $max_b$ where the percentage varies from approx. 20% ($max_b=1$) to 70-80% ($max_b=9$). This demonstrates that, although a small value of $max_b$ would increase $SR_{fl}$ by $\sim 10$ percent points, a small $max_b$ is not desirable since the reduced dataset would contain too few TGFs. Table 3 outlines the total number of TGFs per year, the number of concurrent LIS and ASIM TGF measurements, the number of TGF events associated with the LIS flashes of the reduced dataset for $\Delta t_{TGF-LIS}\le 34$ ms as well as of potential TGF candidates for 34 ms $<\Delta t_{TGF-LIS}\le 100$ ms and 34 ms $<\Delta t_{TGF-LIS}\le 200$ ms. Similar to the starred lines in Fig. 4, it illustrates that the LIS flashes in the reduced dataset can be attributed to associated with approximately 70–80% of the total number of concurrent TGFs per year. In addition to $t_{f,0}, max_b, \Delta t_{pre-main}, r$ and *p*, this can be attributed to two parameters: the time difference $\Delta t_{TGF-LIS}$ and the number of events forming spatial patterns of the LIS data.

Table 3 shows that for 34 ms $<\Delta t_{TGF-LIS}\le 100$ ms, the ratio of the TGF number associated with the LIS flashes in the reduced dataset and the total number of concurrent LIS-TGF measurements is another $\approx 8\%$ percent points; hence, the percentage of coincident LIS-TGF measurements slightly increases with larger $\Delta t_{TGF-LIS}$. This percentage increases more when setting the upper limit of potential TGF candidates to 200 ms. However, we have chosen $\Delta t_{TGF-LIS}=34$ ms as a default value since it cannot be assured that TGFs are actually associated with the correct LIS flash and not a later one when increasing $\Delta t_{TGF-LIS}$, thus giving false positives.

On top of this, a LIS flash is added to the reduced set only if there are sufficient events to identify a distinct spatial pattern and if the spatial pattern lies between triangular and rectangular. Figure 5 shows an example of a lightning event emitting a terrestrial gamma-ray flash, but not being identified by the reduction algorithm. It shows the position of the LIS flash and events, their temporal evolution as well as ASIM optical photograph and photometer plots. Note that LIS measures in a wavelength of 777 nm which is the lowest row in panel f. This figure illustrates that there are too little events whose spatial distribution cannot be identified by the algorithm; this is substantiated by the very weak, noisy ASIM photometer signal in 777 nm. Because of the small number of events, spatially being too close to each other, the reconstruction matrix *R* Eq. 1 consists of only one scalar “1”, i.e. *R* is a $1\times 1$ matrix whose dimensionality is too small to check for the spatial pattern. This issue could be solved by not checking for the spatial pattern of the LIS events as the second step of the algorithm. This would increase the percentage $N_{\text {TGFs,reduced set}}/N_{\text {concurrent LIS-TGF}}$ to approx. 90% compared to $\approx 70-80$%; however, the reduction rate of the flashes would decrease to 2-3% making the reduction algorithm inefficient. Hence, we have decided to not omit the check of the spatial pattern.

**Fig. 6:**
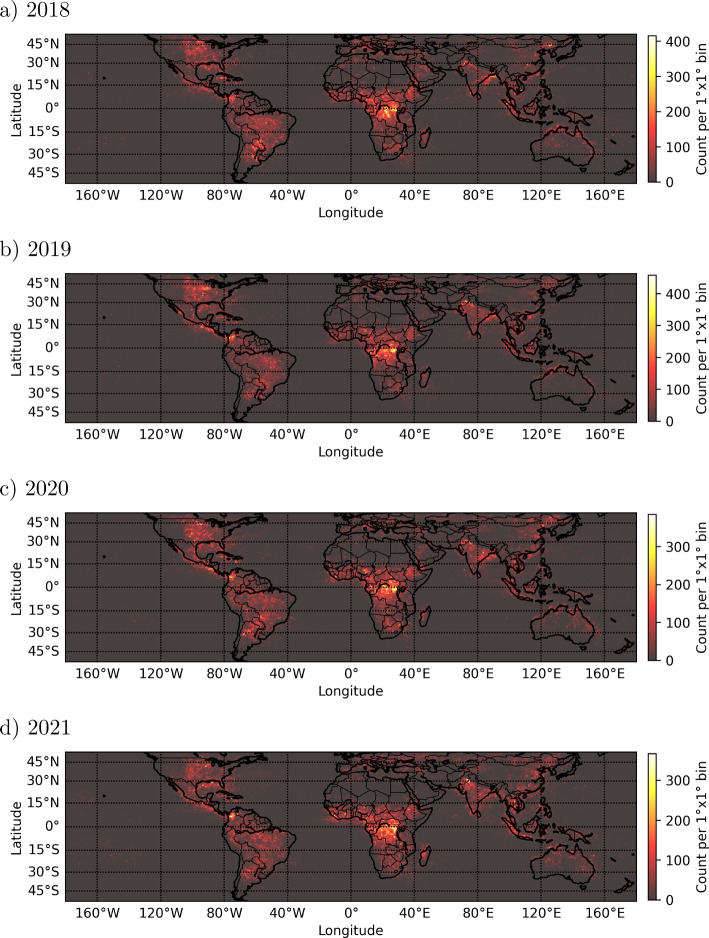
Global distribution of all LIS flashes of the reduced dataset, potentially associated with TGFs

**Fig. 7:**
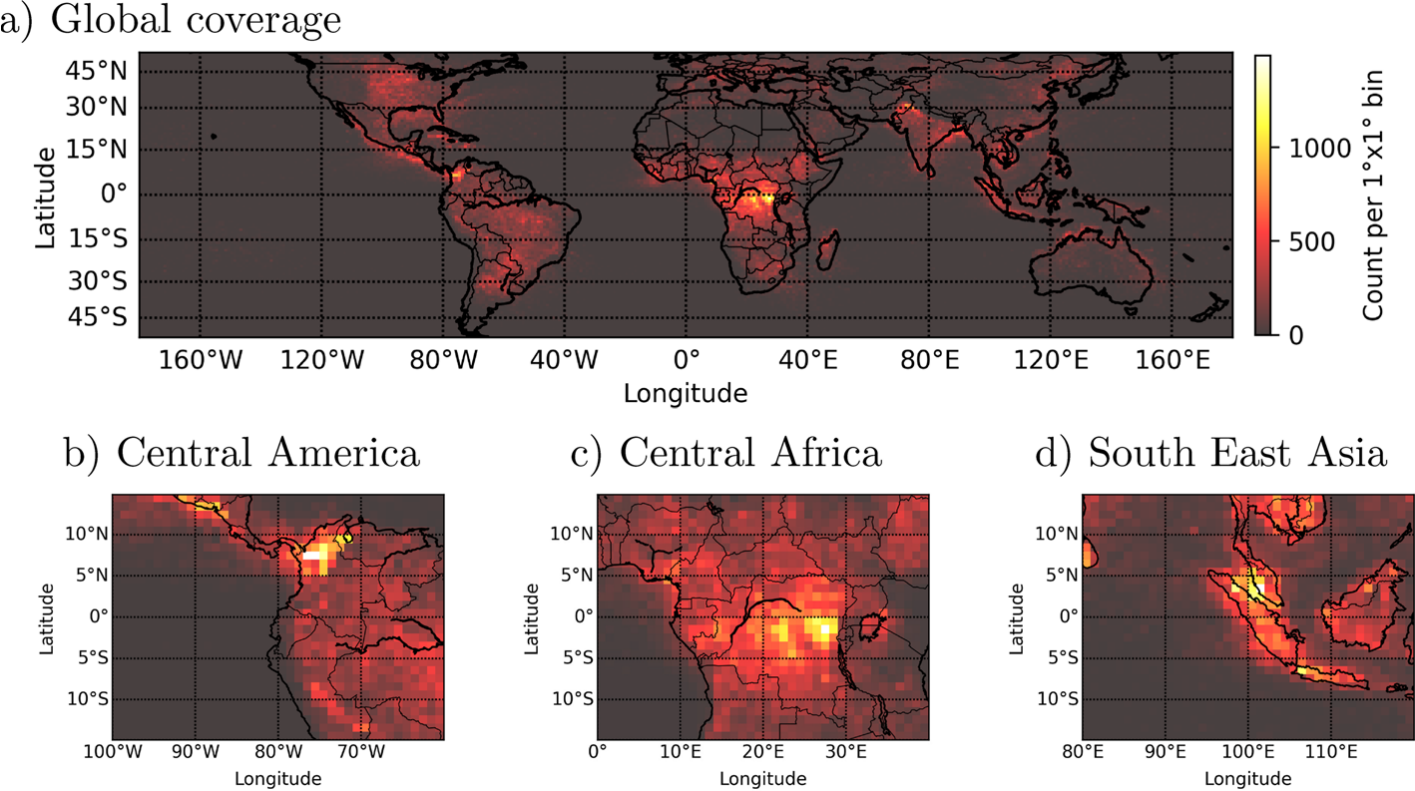
Global distribution of all LIS flashes of the reduced dataset, potentially associated with TGFs, integrated from 2018 until 2021. a) Global coverage, b)-d) Zoom into the main regions of TGF production

**Fig. 8:**
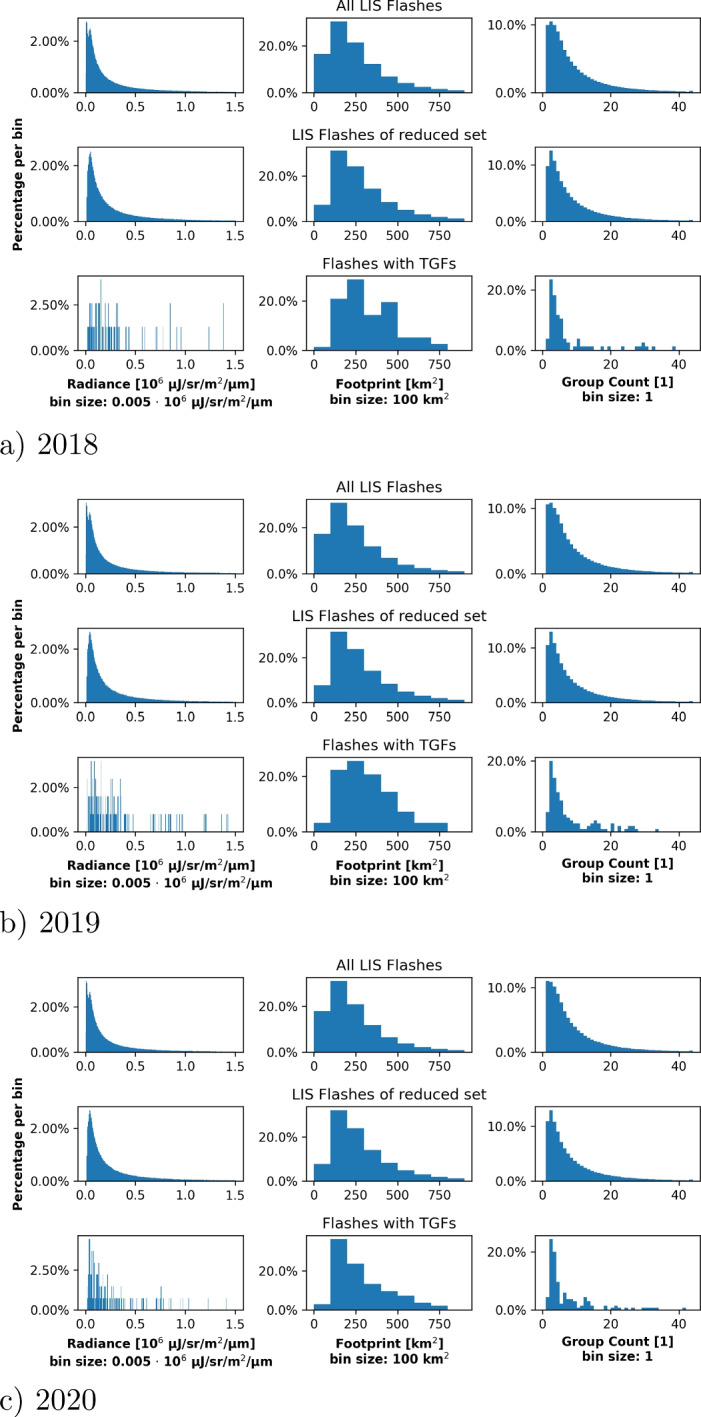
Properties of lightning flashes detected by LIS: The first column shows the distribution of the radiance, the second column of the footprint size and the third column of groups within one flash. The first row in each panel shows the distributions for all detected LIS flashes, the second row the distribution for the reduced dataset and the third one for those flashes confirmed to be associated with TGFs. Figure 9 shows these properties for LIS groups

**Fig. 9:**
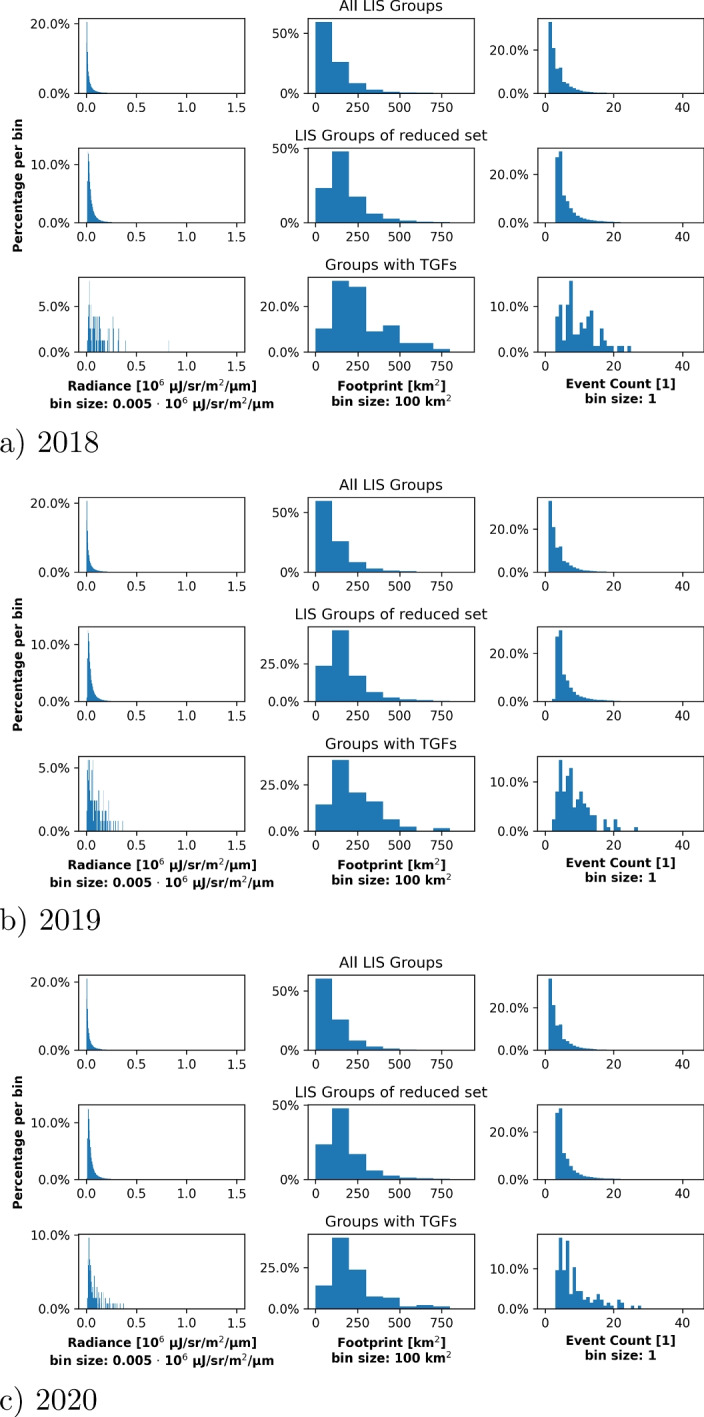
Properties of lightning groups detected by LIS: The first column shows the distribution of the radiance, the second column of the footprint size and the third column of events within one group. The first row in each panel shows the distributions for all detected LIS groups, the second row the distribution for the reduced dataset and the third one for those groups confirmed to be associated with TGFs. Figure 8 shows these properties for LIS groups

**Fig. 10:**
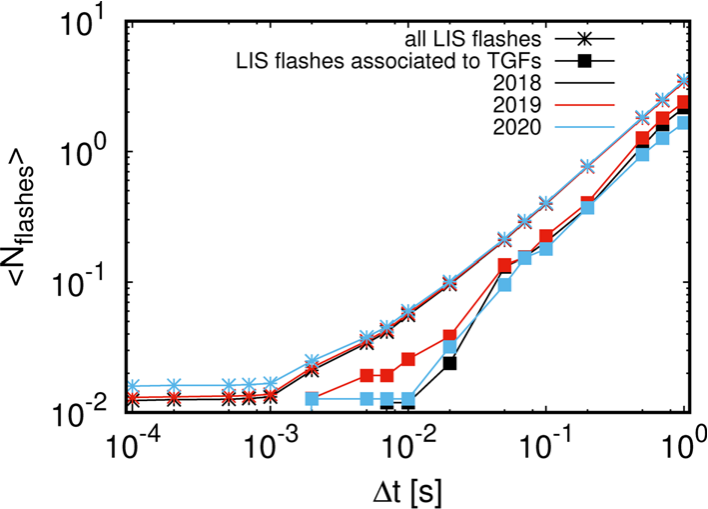
Mean number $\langle N_{flashes}\rangle$ of LIS flashes within a time interval $t\pm \Delta t$ around a given LIS flash for all LIS flashes (line with stars) and for those LIS flashes associated with TGFs (squares). Black lines show data for 2018, red ones for 2019 and blue ones for 2020

### Properties of lightning flashes and groups associated with terrestrial gamma-ray flashes

For the years 2018-2021, Fig. 6 shows the global distribution of all LIS flashes of the reduced dataset, potentially associated with TGFs; Fig. 7 shows the global distribution integrated from 2018 until 2021. For all these years, we can clearly identify the three main lightning/TGF chimneys in Central/Southern America, Central Africa and South East Asia, see Fig. 7 b)-d) (Inan et al. 2006; Carlson et al. 2012; Østgaard et al. 2019b; Maiorana et al. 2021). Especially, in the Americas and in Asia, the appearance of TGFs varies between $+30^{\circ }$ and $-30^{\circ }$ latitude which is also apparent in the reduced datasets. Note that in some rare occasions, TGFs are detected at latitudes of up to $\pm 50^{\circ }$ (Maiorana et al. 2021) which agrees with the extension of the spatial distribution of the LIS flashes shown in Fig. 6.

For the same years, Figs. 8 and 9 show the distribution of the radiance, of the footprint and of the child count (no. of groups per flash and no. of events per group) for all LIS flashes/groups, for the flashes/groups of the reduced set (potentially associated with TGFs) and for the flashes/groups actually associated with TGFs. Both figures show that the considered properties do not vary significantly between all LIS flashes/groups and those associated with TGFs; except for the noisier statistics for the latter one because of the low number of events (cf. Table 3). In all relevant cases, most LIS flashes show a radiance of $\lesssim 0.5\cdot 10^6\ \mu$J/sr/m^2^/$\mu$m where the individual groups show a radiance of $\lesssim 0.2\cdot 10^6\ \mu$J/sr/m^2^/$\mu$m. The maximum footprint size of the LIS flashes is $\sim 800$ km^2^ with a median of $250-300$ km^2^; the maximum footprint size of all LIS groups is $\sim 500$ km^2^ with a median of $100-200$ km^2^. Finally, the number of child counts per flash (group) is mostly below 20. Overall, all these distributions show that there is no significant difference between flashes/groups with or without TGFs and that the radiance, footprint size and child count cannot be used to estimate whether a lightning flash emits a terrestrial gamma-ray flash. Note that this is not contradictory to the presented algorithm which works with the temporal evolution of the LIS groups and the spatial pattern of the LIS events rather than with the radiance, footprint size and child count.

Figure 10 shows the mean number $\langle N_{flashes} \rangle$ of LIS flashes within a time interval $t\pm \Delta t$ around a given LIS flash for all LIS flashes and for those associated with TGFs. It shows that there occur less flashes around a TGF-producing flash than around any average LIS flash which potentially indicates that TGFs are produced by flashes not sharing the available potential with other flashes in the temporal vicinity. This is in alignment with the results by Lindanger et al. (2022) showing that there is significantly less lightning activity up to 150 ms after a TGF.

## Conclusion

We have designed and presented an algorithm to reduce the total number of flashes and groups detected by LIS towards a dataset containing those flashes/groups potentially associated with TGFs. The algorithm is based on two steps which are not influenced significantly by the cloud geometry or the position of the lightning in the cloud or relative to the FOV of the detector: First, the temporal evolution of groups within flashes is determined and those groups which lie within the first 16.2 ms of the flash, have a temporal separation between the pre-activity and the main peak of approximately 5.6 ms and a minimum ratio of 0.22 between these two, are selected as candidate groups. Subsequently, the spatial pattern of all the events framing this candidate group is analyzed and those events forming patterns between triangular and rectangular shape are considered constituents of LIS flashes associated with TGFs. We have performed a parameter study how the reduction effiency depends on the temporal evolution of the group parameters as well as the spatial distribution of the associated events and seen that this algorithm reduced the number of flashes by approximately 60% whilst the number of groups is reduced by approx. 95 %.

The percentage of actual TGFs confirmed to be associated with the LIS flashes of the reduced dataset amounts to 70–80 %. This is partly due to the chosen time difference of 34 ms between the TGF timestamps and the LIS flashes, partly because the spatial pattern of the events of some flashes is not evident enough to pass the algorithm. Increasing $\Delta t_{TGF-LIS}$ allows to increase the percentage of TGFs associated with LIS flashes of the reduced dataset and of concurrent LIS and TGF measurements; however, this also increases the chance for false coincidences when a LIS flash is actually not associated with a TGF. Not checking the spatial pattern is not feasible as this would decrease the reduction efficiency of LIS flashes down to $2-3\%$.

Finally, we presented a comparison of the distribution of the radiance, the footprint size and the child count between all LIS flashes/groups, those from the reduced dataset that are potentially associated with TGFs, and those that are actually associated with TGFs. In our analysis, we have not found any significant difference amongst these three categories concluding that lightning flashes emitted terrestrial gamma-ray flashes are not special with respect to radiance, footprint size and child count. However, these are not relevant input parameters for the presented algorithm and thus do not enter it at all. Instead, we found that TGF producing LIS flashes have less flashes in their temporal proximity indicating that flashes associated with TGFs might not share the available electric potential with other LIS flashes. However, we leave a further analysis of this observation for future work since the scope of this paper is to present an algorithm helping to identify LIS flashes related to TGFs.

As LIS detects approximatey $10^6$ lightning flashes per year, the manual check for associated TGFs might be a tremendous workload. In the future, the presented algorithm will help to make a pre-selection of which lightning flashes to analyze for the simultaneous occurrence of TGFs. We emphasize that the presented algorithm is currently applicable for space-based measurements of lightning flashes and validated by TGFs observed from ASIM. However, there have been measurements of lightning and TGFs through aircraft missions (Smith et al. 2011; Kochkin et al. 2015, 2018) allowing to measure more TGFs than space-based measurements since the absorption of TGF photons in the atmosphere is mitigated. In the future, we expect to be more such missions such as the concurrent “Airborne Lightning Observatory for FEGS and TGFs” (ALOFT) (for more information, see Østgaard et al. (2023a)) indicating that thunderstorms might produce more TGFs than previously thought Østgaard et al. 2023b). Such missions will help us to verify that the assumptions for the satellite-based algorithm also hold for detections at thunderstorm altitudes. Finally, the algorithm, which can be obtained from https://gitlab.gbar.dtu.dk/chrstk/lis-tgf-algorithm.git, is to be developed further to be compatible with measurements at thunderstorm altitudes such that it will be of use for a wider collection of lightning and TGF measurements.

## Data Availability

Data from ISS LIS can be downloaded from https://ghrc.nsstc.nasa.gov/pub/lis/iss/data/science/ Lang (2022). ASIM data can be obtained from the ASIM Science Data Center (ASDC), https://asdc.space.dtu.dk/.

## Appendix A Derivation of matrix sums

We here give a brief derivation of the matrix pattern sums summarized in Table 2.

Let us start with a rectangular pattern of an $m\times n$ matrix

A1 $$\begin{aligned} R=\left. \underbrace{\left( \begin{array}{cccc} 1 &{} 1 &{} \ldots &{} 1 \\ 1 &{} 1 &{} \ldots &{} 1 \\ \vdots &{} \ddots &{} \ddots \vdots \\ 1 &{} 1 &{} \ldots &{} 1 \end{array} \right) }_{n\ \text {elements}} \right\} m\ \text {elements} \end{aligned}$$

such that *R* as in Eq. 1 consists of only ones. The convolution with the kernel $$\left( \begin{array}{cc} 1 &{} 1 \\ 1 &{} 1 \end{array} \right) $$ leads to

A2 $$\begin{aligned} R\times \left( \begin{array}{cc} 1 &{} 1 \\ 1 &{} 1 \end{array} \right) = \left( \begin{array}{cccc} 4 &{} 4 &{} \ldots &{} 4 \\ 4 &{} 4 &{} \ldots &{} 4 \\ \vdots &{} \ddots &{} \ddots \vdots \\ 4 &{} 4 &{} \ldots &{} 4 \end{array} \right) = 4 \left. \underbrace{\left( \begin{array}{cccc} 1 &{} 1 &{} \ldots &{} 1 \\ 1 &{} 1 &{} \ldots &{} 1 \\ \vdots &{} \ddots &{} \ddots \vdots \\ 1 &{} 1 &{} \ldots &{} 1 \end{array} \right) }_{(n-1)} \right\} (m-1) \end{aligned}$$

such that the sum over all entries of the convolution amounts to $\sigma _{m,n,\Box }=4\cdot (m-1)(n-1)$. In the case of a rectangle without corners Eq. 1 looks liks

A3 $$\begin{aligned} \left. \underbrace{\left( \begin{array}{cccc} 0 &{} 1 &{} \ldots &{} 0 \\ 1 &{} 1 &{} \ldots &{} 1 \\ \vdots &{} \ddots &{} \ddots \vdots \\ 0 &{} 1 &{} \ldots &{} 0 \end{array} \right) }_{n\ \text {elements}} \right\} m\ \text {elements} \end{aligned}$$

such that the zeros in the corners condition the sum of the convolution to be 4 units less, thus through the zeros in the corners, thus $4\cdot (m-1)(n-1)-4$.

Now let us assume a triangular pattern

A4 $$\begin{aligned} \left. \underbrace{\left( \begin{array}{ccccccc} 1 &{} 1 &{} 1 &{} \ldots &{} 1 &{} 1 &{} 1 \\ 0 &{} 1 &{} 1 &{} \ldots &{} 1 &{} 1 &{} 1 \\ \vdots &{} \ddots &{} \ddots &{} \ddots &{} \ddots &{} \vdots \\ 0 &{} 0 &{} 0 &{} 0 \ldots &{} 0 &{} 1 &{} 1 \\ 0 &{} 0 &{} 0 &{} 0 \ldots &{} 0 &{} 0 &{} 1 \end{array} \right) }_{n\ \text {elements}} \right\} m\ \text {elements} \end{aligned}$$

such that the convolution becomes

A5 $$\begin{aligned} \left( \begin{array}{ccccccc} 1 &{} 1 &{} 1 &{} \ldots &{} 1 &{} 1 &{} 1 \\ 0 &{} 1 &{} 1 &{} \ldots &{} 1 &{} 1 &{} 1 \\ \vdots &{} \ddots &{} \ddots &{} \ddots &{} \ddots &{} \vdots \\ 0 &{} 0 &{} 0 &{} 0 \ldots &{} 0 &{} 1 &{} 1 \\ 0 &{} 0 &{} 0 &{} 0 \ldots &{} 0 &{} 0 &{} 1 \end{array} \right) * \left( \begin{array}{cc} 1 &{} 1 \\ 1 &{} 1 \end{array} \right) = \left. \underbrace{\left( \begin{array}{ccccccc} 3 &{} 4 &{} 4 &{} \ldots &{} 4 &{} 4 &{} 4 \\ 1 &{} 3 &{} 4 &{} \ldots &{} 4 &{} 4 &{} 4 \\ \vdots &{} \ddots &{} \ddots &{} \ddots &{} \ddots &{} \vdots \\ 0 &{} 0 &{} 0 &{} 0 \ldots &{} 1 &{} 3 &{} 4 \\ 0 &{} 0 &{} 0 &{} 0 \ldots &{} 0 &{} 1 &{} 3 \end{array} \right) }_{(n-1)} \right\} (m-1). \end{aligned}$$

The sum over the elements of the convolved matrix has three contributions:

- The diagonal elements are always 3 and appear $\min (m-1,n-1)=\min (m,n)-1$ times, hence $3\left( \min (m,n)-1\right)$.
- The same logic applies for the off-diagonal elements one element left of the diagonal elements. Here, the entry is always 1 and there is one element less than for the diagonal elements, thus in total $1\left( \min (m,n)-1-1\right) =\min (m,n)-2$.
- The elements right off the diagonal are all 4. From bottom to top, the 4 appears $0,1,2,\dots \min (m,n)-1$ times, hence $4\cdot \sum \limits _{j=1}^{\min (m,n)-1} j$.

Conclusively, the pattern sum for triangles amounts to $\sigma _{m,n,\triangle }=3\left( \min (m,n)-1\right) +\min (m,n)-2 +4\cdot \sum \limits _{j=1}^{\min (m,n)-1} j$.

## Appendix B Details of the algorithm code

We here describe in detail how the algorithm code works and which output files are generated.

The algorithm consists of three files, tgf_algo_read_ncdf.py, lis.py and file_management.py. The latter two are for file I/O whilst the main algorithm is encoded in tgf_algo_read_ncdf.py.

In tgf_algo_read_ncdf.py, the input folder with LIS source data (stored as Network Common Data Format (netCDF) files) is specified in line 580 (storage_folder) whilst the output folder result_folder is specified in line 40. Note that for each day per year, the input LIS data is to be stored in subfolders of the type yy-mm-dd where yy, mm and dd denote the two-digits year, month and day. The function to determine the candidate group starts in line 110 (select_TGF_candidate_group), based on the algorithm presented in Section 2.2.1. The selected groups are passed to the function convolution_to_select (line 261) checking the spatial pattern of the events constituing these groups, see Section 2.2.2. In line 583, the function mbsyyyy=STIL.generate_month_boundaries(yyyy) is called which generates the day boundaries for each month in the year yyyy. The actual algorithm is called through download_errors, file_errors = STIL.main(mbsyyyy, storage_folder, skip_download=True, overwrite_output_files=True) (lines 588–589). mbsyyyy transfers the month boundaries and storage _folder the directory of saving the output files. If the input LIS data is available, skip_download needs to be set to True; overwrite_output_files determines whether output are allowed to be overwritten.

While the code running, it creates four files per month *mm* for the given year *yyyy*:

1. *yyyy*-*mm*_AllLISFlashes_ParameterSetting_V0.1.txt: This file lists all LIS flashes per month and year, hence before applying the algorithm. In ascending order of columns, the file includes: the date and time of the flash, its latitude, longitutde, radiance, footprint size, child count (i.e. number of groups) as well as the time, latitutde, longitude, radiance, footprint size and child count (number of events) for the first group of that flash.
2. *yyyy*-*mm*_AllLISGroups_ParameterSetting_V0.1.txt: The structure of this file is the same as for *yyyy*-*mm* _AllLISFlashes_ParameterSetting_V0.1.txt, but shows values for all groups of a flash, not only the first one.
3. *yyyy* -*mm*_AssertionErrorsDuringAlgorithm_ParameterSetting_V0.1.txt: This file summarizes the files which could not be handled by the presented algorithm.
4. *yyyy*-*mm*_PotentialTGFs_ParameterSetting_V0.1.txt: This file shows the same information as *yyyy*-*mm* _AllLISFlashes_ParameterSetting_V0.1.txt, but only for the LIS flashes of the reduced dataset, i.e. after applying the algorithm and hence potentially being associated with the occurrence of TGFs. In addition, the last five columns mark with a “1” whether the spatial pattern is quadratic, rectangular, triangular, cornerless rectangular or other. In *yyyy*-*mm* _AllLISFlashes_ParameterSetting_V0.1.txt and *yyyy*-*mm*_AllLISGroups_ParameterSetting_V0.1.txt, these values are set to “0” by default as it is not necessary to define the spatial pattern for all LIS flashes and groups.
